# Role and regulation of surface oxygen vacancies in vanadium-based oxides for chemical looping oxidative dehydrogenation of propane†

**DOI:** 10.1039/d4sc07811j

**Published:** 2025-01-22

**Authors:** Dehui Luo, Ran Luo, Xianhui Wang, Xin Chang, Tingting Yang, Sai Chen, Zhi-Jian Zhao, Jinlong Gong

**Affiliations:** a School of Chemical Engineering & Technology, Key Laboratory for Green Chemical Technology of Ministry of Education, Tianjin University, Collaborative Innovation Center for Chemical Science & Engineering (Tianjin) Tianjin 300072 China zjzhao@tju.edu.cn jlgong@tju.edu.cn; b Joint School of National University of Singapore and Tianjin University, International Campus of Tianjin University Binhai New City Fuzhou 350207 Fujian China; c International Joint Laboratory of Low-Carbon Chemical Engineering Tianjin 300192 China; d Haihe Laboratory of Sustainable Chemical Transformations Tianjin 300192 China; e National Industry-Education Platform of Energy Storage, Tianjin University 135 Yaguan Road Tianjin 300350 China; f Tianjin Normal University Tianjin 300387 China

## Abstract

Redox catalysts play a critical role in chemical looping oxidative dehydrogenation of propane (CL-ODH). However, challenges persist in modulating lattice oxygen in metal oxides and maintaining surface oxygen coverage to prolong the oxidative dehydrogenation stage. This paper describes the role of oxygen vacancies by evaluating numerous vacancy distribution patterns, including surface and bulk distributions, to identify VO_*x*_ surfaces across a wide range of reduction degrees, guided by calculated oxygen vacancy formation energy. The surface reactions are classified into three distinct stages based on surface oxygen vacancy coverage (Ovc), with transitions between stages attributed to the excessive reactivity of lattice oxygen, variations in vanadium valence states, and the localized limitations of vacancy effects. Additionally, four high-valent metal dopants (W, Mo, Nb, and Os) identified through charge transfer energy (CTE)-based descriptors effectively reduce oxygen reactivity while optimizing the utilization of bulk lattice oxygen to maintain favorable surface Ovc. These findings provide essential theoretical insights and a strategic framework for the rational design of redox catalysts in CL-ODH applications.

## Abstract 2

Redox catalysts play a critical role in chemical looping oxidative dehydrogenation of propane (CL-ODH).

## Graphical abstract

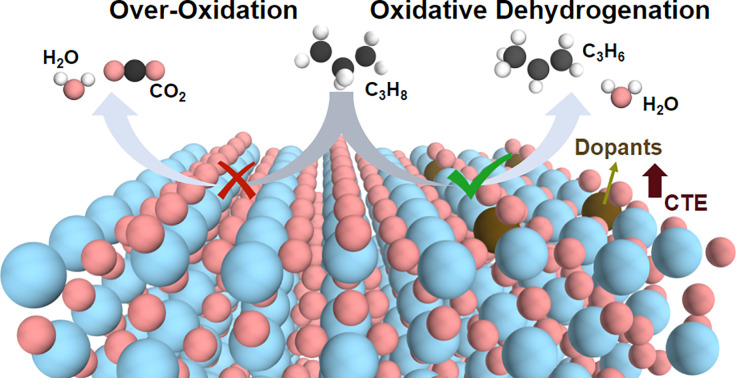

## Introduction

The increased availability of low-cost propane, driven by the shale gas revolution, has generated significant interest in propane dehydrogenation.^1–4^ However, current industrial catalysts for nonoxidative propane dehydrogenation (PDH) face limitations due to their endothermic nature (Δ_r_*H*° = 124 kJ mol^−1^) and the high operating temperatures (>500 °C) required for viable propane conversion (∼40%), which lead to severe catalyst deactivation from coking.^5–8^ Alternative oxidative dehydrogenation (ODH) offers a potential solution by introducing an oxidant to overcome thermodynamic constraints and suppress coking.^9–12^ Nonetheless, high selectivity is often compromised by consecutive oxygenation reactions, and co-feeding with gaseous molecular oxygen poses explosion risks.^13–15^

In contrast, chemical looping oxidative dehydrogenation of propane (CL-ODH) technology employs redox catalysts, typically referred to as oxygen carriers, to facilitate the cyclic transfer of matter and energy within the system.^16–18^ In this process, lattice oxygen from metal oxides serves as the sole oxidant during dehydrogenation, with controlled utilization ensuring a moderately selective ODH process and significantly reducing the explosion risk associated with molecular oxygen co-feeding.^19^ Modeling predicts a maximum propylene yield of 73.4%, presenting highly attractive performance even compared to existing ODH technologies.^20^ Vanadium oxides have been extensively investigated as efficient catalysts for both PDH and ODH.^21–23^ Nevertheless, their application in CL-ODH is hindered by over-oxidation, as high valence vanadium (V^5+^) tends to oxidize propane into CO_*x*_ rather than selectively producing propylene.^24,25^ V^4+^ species have been identified as the optimal phase for moderate activity and selectivity, whereas V_2_O_3_ served as unreducible PDH species.^26–28^ Recently, high-loading vanadium-based oxides (40 wt% VO_*x*_/Al_2_O_3_) have exhibited promising catalytic activity, achieving a 36% propane conversion rate at 500 °C, coupled with rapid lattice oxygen diffusion occurring within minutes during the reaction stages, while displaying distinct stage-wise reaction characteristics.^24,27,29,30^ However, it remains unclear which oxygen species are responsible for selective oxidative dehydrogenation and the selectivity of propylene in CL-ODH still shows certain disparities compared to that in PDH. Additionally, the distinction between ODH and PDH, as well as strategies to extend the lifetime of the ODH stage, remains to be further investigated.

Oxygen vacancies in reducible oxide oxygen carriers are key factors in regulating their catalytic performance. Fan *et al.*^31^ demonstrated that in the chemical looping methane conversion process, oxygen vacancies facilitate partial oxidation by lowering dehydrogenation barriers, although excessive vacancy concentration limits further catalytic improvement. Chen *et al.*^32^ found that changes in surface oxygen concentration drive the shift of perovskite oxides from complete combustion (O-rich surfaces) to selective oxidation (O-deficient surfaces), with vacancy concentration tunable by controlling the oxidation time. Additionally, our previous work^27,33^ identified that the oxygen reactivity was dependent on the dispersion states of supported VO_*x*_, with activity increasing as V loadings increase. However, the role of oxygen vacancies as key modulators of catalytic performance remains unresolved. Hence, revealing the structure–performance relationship between the reduction process of the oxygen vacancies and catalytic performance is of vital importance, particularly for the rational design of redox catalysts that transcends traditional trial-and-error methods.

In this work, using density functional theory (DFT), nearly a thousand possible vacancy distribution patterns were calculated to elucidate the redox processes of VO_*x*_-based oxygen carriers, identifying surfaces with varying oxygen vacancy coverage (Ovc) through a stepwise oxygen reduction method. Reaction network analysis categorized the surface reactions into three distinct stages based on Ovc, with stage transitions attributed to the excessive reactivity of lattice oxygen, fluctuations in vanadium valence states, and localized vacancy effects, as revealed by electronic and geometric structure analyses. High-valent metals (W, Mo, Nb, and Os), identified *via* improved charge transfer energy (CTE)^34^-based descriptors which describe the electron-donating ability of lattice oxygen, effectively reduced C–H bond activation capacity while maintaining surface Ovc thus extending the ODH stage lifetime. These findings provide valuable insights for screening efficient oxygen carriers and optimizing C–H bond activation to achieve more efficient catalytic processes.

## Results and discussion

### VO_*x*_-based oxygen carrier reduction process

**Fig. 1:**
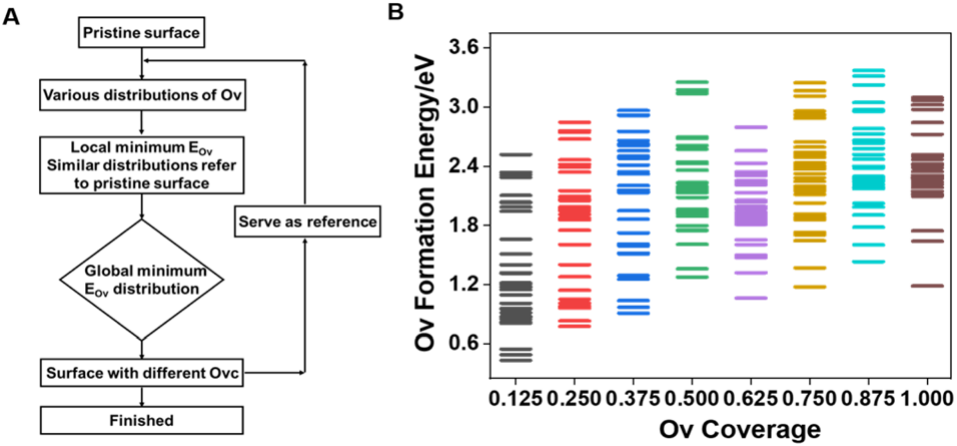
Scheme of the stepwise reduction approach for removing lattice oxygen species (A). Ov formation energy of VO_*x*_ surfaces with different Ovc across numerous Ov distribution patterns (B).

To unravel the reduction process and construct a series of VO_*x*_ surface models with varying Ovc, we systematically removed lattice oxygen from a 4 × 4 expansion of the VO_2_ slab using a stepwise reduction method (Fig. 1A), and V^4+^ species in VO_2_ showed the best performance with balanced oxygen vacancy (Ov) formation energy and propylene adsorption energy compared to those in V_2_O_5_ and V_2_O_3_ (Fig. S1†). Coverage refers to the ratio of oxygen vacancies in the defective surface to the active oxygens in the pristine surface. The optimal Ov distribution pattern, reflecting the redox properties of lattice oxygen, was determined based on the magnitude of oxygen vacancy formation energy (eqn (1)),^35,36^ with full computational details provided in the ESI.†

1 *E*_Ov_ = *E*_(1+*x*)Ov_ − *E*_*x*Ov_ + [C_3_H_6_ (g) + H_2_O (g) − C_3_H_8_ (g)]

Here, *E*_(1+*x*)Ov_ and *E*_*x*Ov_ represent the energy for the relaxed slab with 1 + *x* and *x* oxygen vacancies, respectively. C_3_H_6_ (g) + H_2_O (g) − C_3_H_8_ (g) was used as a reference for single oxygen energy.

As shown in Fig. 1B, a wide range of potential multi-oxygen vacancy models were examined, with nearly a thousand vacancy distribution patterns calculated, encompassing surface and bulk distributions. The locations of optimally reducing oxygen species, defined by the lowest *E*_Ov_ across different Ovc levels, are illustrated in Fig. S2.† Among all investigated Ov distribution patterns for the pure VO_*x*_ system, surface bridge oxygen species consistently displayed higher redox activity. During chemical looping, bulk lattice oxygen diffuses to the surface to replenish vacancies, with *E*_Ov_ serving as a thermodynamic descriptor for diffusion tendency. Bulk lattice oxygen generally has higher vacancy formation energy than surface bridge oxygen. Furthermore, oxygen vacancies exhibited a strong tendency to cluster, which was energetically favorable, lowering both individual and average Ov formation energy across the entire investigated Ovc range (Fig. S3†). Thermodynamically, oxygen vacancies primarily concentrate on the surface and aggregate, contributing to a more stable overall structure.

### Reaction mechanisms

**Fig. 2:**
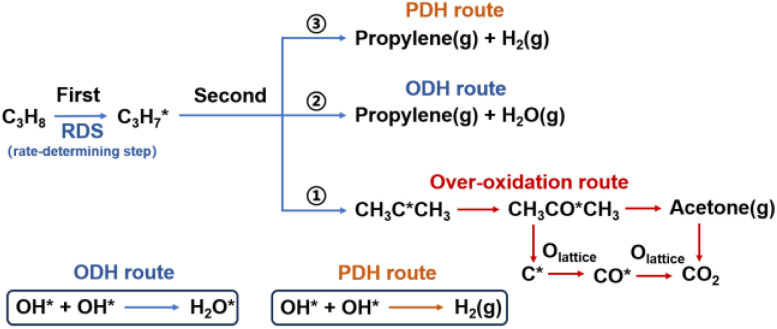
Scheme of reaction routes during the CL-ODH process.

**Fig. 3:**
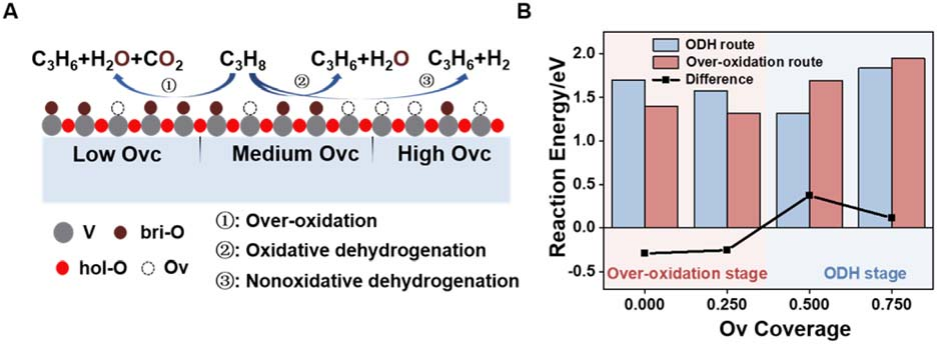
Three distinct reaction stages determined by surface Ovc (A). Plot of different Ovc surfaces against the second C–H bond activation reaction energy of the 
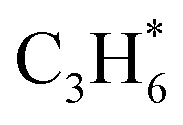
 route and CH_3_O*CH_3_ route (B).

**Fig. 4:**
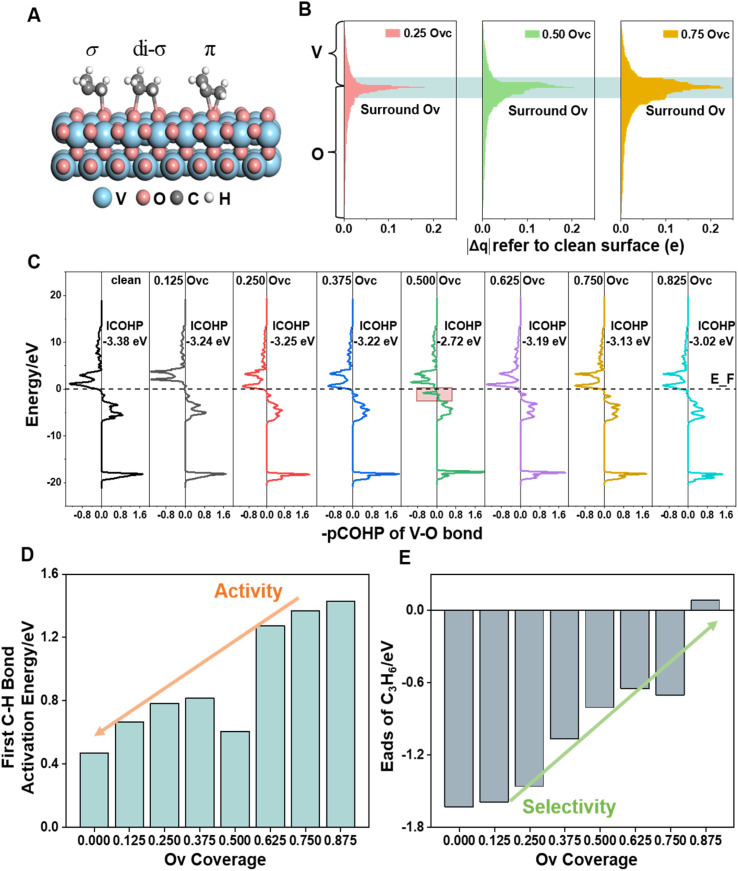
Adsorption modes of propylene on VO_*x*_ surfaces (A). Bader charge analysis; the absolute charge difference for each remaining ion, referenced to the pristine surface site upon oxygen vacancy formation, is shown, with data for the nearest neighbouring transition metals and lattice oxygen indicated in light blue shading (B). The –pCOHP curves for V–O bonds within V–O–V species (focusing on the V–O bond with the lower ICOHP value) across the calculated range of Ovc surfaces, with data for unique antibonding of the 0.50 Ovc surface V–O bond indicated in red shading (C). Plot of different Ovc surfaces against the first C–H bond activation energy (D) and (E) propylene adsorption energy. The data shown represent the sites with the strongest activity and strongest adsorption, respectively.

During the CL-ODH process, redox catalysts demonstrate distinct stage-specific reaction characteristics.^37–39^ The activation of alkanes during the dehydrogenation process occurs *via* lattice oxygen within carriers, rather than through gas-phase molecular oxygen. The evolution of lattice oxygen alters the surface Ovc, subsequently influencing the reaction route. As illustrated in Fig. 2, these routes include over-oxidation, oxidative dehydrogenation (ODH), and propane dehydrogenation (PDH) mechanisms. To elucidate the reaction mechanisms on surfaces with different Ovc, we first performed a comparative analysis of the H_2_ formation route [2OH* → H_2_] against the H_2_O formation route [2OH* → H_2_O* + Ov]. In this work, H_2_O is produced *via* the ODH route, while H_2_ is generated through the PDH route.^40^ As illustrated in Fig. S4,† as Ovc increases, the H_2_O formation route with lower reaction energy compared to the H_2_ formation route, indicating the reaction stage transitions from the ODH stage to the PDH stage. Moreover, the continuous oxidation of propane is the main factor contributing to the lower selectivity of ODH compared to PDH. Previous studies have established that adsorbed acetone (CH_3_O*CH_3_) is the key intermediate responsible for over-oxidation.^41–43^ We systematically compared the reaction energies associated with the formation of 
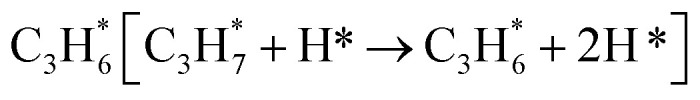
 and 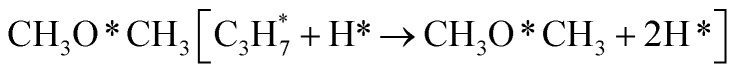 routes. As shown in Fig. 3B, the CH_3_O*CH_3_ route on pristine and initially reduced VO_*x*_ surfaces (Ovc range: 0.0–0.50) exhibited lower reaction energy, corresponding to the over-oxidation stage. With further consumption of lattice oxygen (Ovc range: 0.50–0.75), the 
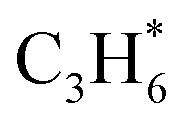
 route displayed lower reaction energy compared to the CH_3_O*CH_3_ route. Subsequent analysis indicated that surfaces within this range also exhibited lower propylene adsorption energy (Fig. 4E), highlighting the importance of effective propylene desorption to suppress further oxidation.^14,44,45^ Therefore, a medium Ovc range is favorable for propylene generation through the ODH route, aligning with optimal surface vacancy coverage. Overall, the surface reaction can be classified into three stages based on different Ovc (Fig. 3A):

Stage I: over-oxidation (at low Ovc)

2 C_3_H_8_ + 10O (lattice oxygen) → 3CO_2_ + 4H_2_O + 10Ov

Stage II: oxidative dehydrogenation (at medium Ovc)

3 C_3_H_8_ + O (lattice oxygen) → C_3_H_6_ + H_2_O + Ov

Stage III: nonoxidative dehydrogenation (at high Ovc)

4 C_3_H_8_ → C_3_H_6_ + H_2_

Here, low Ovc denotes the 0–25% ratios of oxygen vacancy compare to the pristine surface, medium Ovc denotes the 25–75% ratios of oxygen vacancy compare to the pristine surface, and high Ovc denotes the 75–100% ratios of oxygen vacancy compare to the pristine surface.

The over-oxidation stage in the pure vanadium oxides is excessively prolonged, accounting for approximately half of the process, whereas the ODH stage is comparatively brief, constituting only about one-quarter. This underscores the necessity of regulating surface Ovc to maintain it within an optimal range to extend the duration of the ODH stage lifetime and improve catalytic performance.

### Role of the vacancy effect

For revealing the motivation behind the reaction stage transition, Bader charge, density of state (DOS) and Crystal Orbital Hamilton Population (COHP) analyses were performed on surfaces with varying Ovc to understand the geometric and electronic structure effects of vacancies. Following surface relaxation, adjacent metals near the Ov tended to form metal–metal bonds (3.04 Å → 2.81 Å) to achieve a lower energy state, with the distortion induced by the vacancy localized around it (Fig. S5†). Bader charge analysis was performed to quantify the charge redistribution around each atom.^46^ As depicted in Fig. 4B and S6,† most excess electrons were absorbed by adjacent sites, particularly affecting the valence state of the neighboring vanadium atoms. The average valence state of surface vanadium decreased from 1.90e to 1.74e, where the transition from 1.90e to 1.79e corresponds to the over-oxidation stage, the range from 1.79e to 1.74e reflects the ODH stage, and values below 1.74e indicate the PDH stage. Additionally, the electronic structure effects induced by the vacancy were found to be delocalized,^47^ as revealed by electronic structure and charge density difference analyses (Fig. S7†). The COHP technique was employed to quantify bonding strength and provide insights into bonding characteristics through orbital interactions.^48^ The –COHP curves (Fig. 4C and S8†) showed that at 0.50 Ovc, the presence of antibonding states below the Fermi level suggested V–O bond instability, correlating with enhanced C–H activation. Thus, while surface vacancies significantly alter the local electronic and geometric environments of adjacent sites, their effects on the valence states and bonding characteristics of neighboring metal atoms are less pronounced due to the delocalized nature of the vacancy effect.

Building upon previous research, vacancies in redox catalysts play a critical role in reactions by altering electronic and geometric structures or serving as potential active sites.^49–51^ Hydrogen atom affinity reflects site activity.^52^ Additionally, vacancies create a complex surface microenvironment comprising various sites,^53,54^ motivating investigation into all possible reaction sites on different Ovc surfaces. Our calculations accounted for all potential adsorption sites, including vacancies and lattice oxygen species on VO_*x*_ surfaces. As shown in Fig. S9C,† hydrogen atoms exhibited stronger adsorption on bridge oxygen sites than on Ov sites across the examined Ovc range, with the strongest adsorption energy being −1.32 eV for bridge oxygen sites and the weakest being 1.51 eV for Ov sites. This finding suggests that, for the first dehydrogenation step 
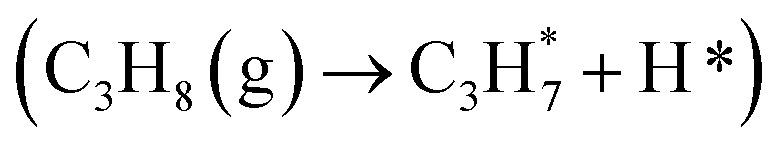
, lattice oxygen serves as a more favorable active site compared to oxygen vacancies. Furthermore, we compared the C–H bond activation energy, H adsorption energy, and Ov formation energy of different oxygen species (bridge oxygen and hollow oxygen) on the pristine surface (Fig. S9D†). The results revealed that bridge oxygen is more likely to serve as the active oxygen species during the reaction. In the ODH process, the rate-determining step (RDS) may be either the first or second dehydrogenation step 
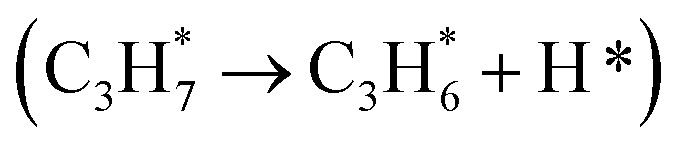
.^55^ Our free energy calculations for these steps (Fig. S10C†) identified the first dehydrogenation step as the RDS, with Δ*G*_1_ = 1.91 eV and Δ*G*_2_ = 1.39 eV. Accordingly, subsequent discussions are based on the first dehydrogenation step being the RDS.

To explore the relationship between surface activity and vacancy coverage, we conducted kinetic calculations on all potential dehydrogenation sites across the full spectrum of Ovc. As shown in Fig. 4D, the capacity for C–H bond activation is particularly high on the initially reduced surfaces and decreases gradually as Ovc increases. The 0.50 Ovc surface exhibited unique C–H activation characteristics due to its geometry and the unstable V–O bond (Fig. 4C). Propylene desorption is crucial for enhancing the selectivity of propane dehydrogenation, prompting a comprehensive exploration of various adsorption configurations of propylene in this work (Fig. 4A and S9†). Within the investigated Ovc range, propylene showed the strongest adsorption in the dioxygen configuration (di-σ) on the VO_*x*_ surfaces. Fig. 4E illustrates that propylene adsorption is notably strong on initially reduced surfaces but diminishes with increasing Ovc, demonstrating an inverse relationship compared to activity. This trend is particularly significant as it highlights the inherent trade-off between reaction activity and selectivity regulated by oxygen vacancies, a characteristic feature of redox-type metal oxide catalysts. This balance arises from the dual role of vacancies, which enhance selectivity while simultaneously reducing lattice oxygen reactivity. Understanding and optimizing this trade-off is critical for improving the catalytic performance and achieving a desired favorable yield in redox catalysts.

Overall, oxygen vacancies serve as important point defects that regulate both the selectivity and activity of vanadium-based oxides during the dehydrogenation process. However, the high reactivity of lattice oxygen and the delocalization effects of vacancies necessitate consuming a sufficient amount of lattice oxygen to maintain surface reactions within the ODH stage. Furthermore, design strategies such as defect engineering—controlling the oxidation time of oxygen carriers to adjust defect concentration—must navigate the trade-off between reaction activity and selectivity.

### Regulation of surface Ov coverage

**Fig. 5:**
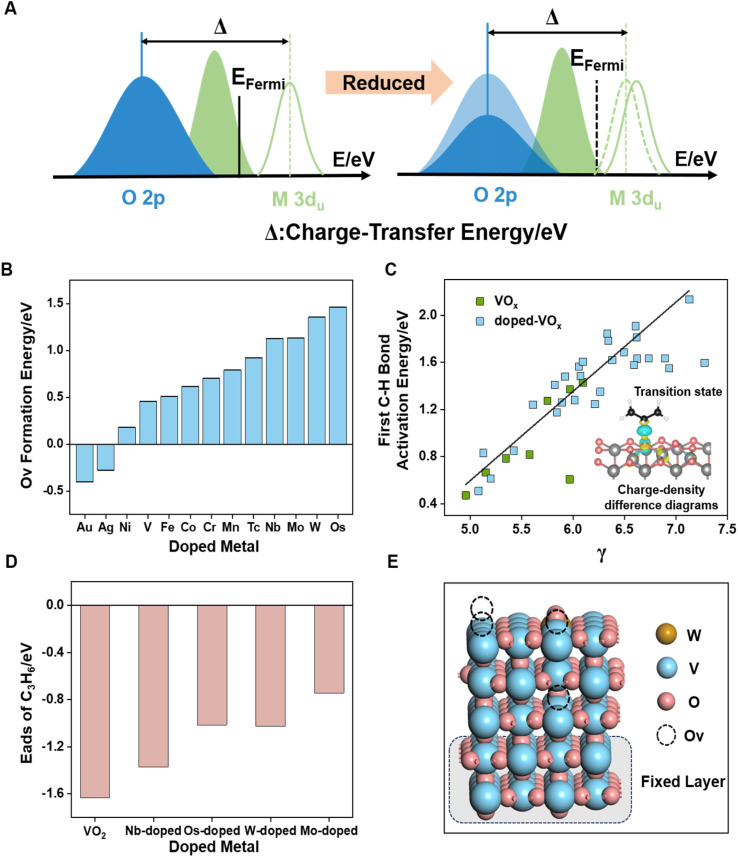
Schematic diagrams (A) of the orbital charge-transfer energy (left) and the effect of oxygen vacancy on charge-transfer energy (right). Ov formation energy corresponding to different metal dopants (B). Plot of *γ* against the first C–H bond activation energy (C), and the inset shows the charge density difference of the radical-like transition state; yellow indicates e^−^ donation and blue indicates e^−^ acceptance. Propylene adsorption energy corresponding to different metal-doped VO_*x*_ surfaces (D). Distribution pattern of the W-doped VO_*x*_ slab model coexisting with bulk and surface oxygen vacancies (E).

Upon the formation of oxygen vacancies on metal oxide surfaces, as shown in Fig. 5A, electrons transfer from the O 2p-band center to the Fermi energy (*E*_Fermi_), causing a shift in the O 2p-band center away from *E*_Fermi_. Concurrently, the unoccupied 3d-band center of the metal coordinated with oxygen moves closer to *E*_Fermi_, indicating that C–H bond activation depends on both the properties of lattice oxygen and the coordinated metal. Charge density difference (Fig. 5C inset) of the radical-like transition state of C–H bond activation revealed that the electron transfer process involves both the coordinated metal and lattice oxygen. Based on the observed changes in metal valence and Ovc, we devised a C–H bond activation descriptor using charge transfer energy (CTE).^34,56^ The CTE refers to the difference between the O 2p-band center and the unoccupied metal d-band center (eqn (5)). To account for the variation in Ovc across different VO_*x*_ surfaces, the CTE was further refined to develop a novel parameter, denoted as *γ* (eqn (6)).

5 CTE = average coordinated metals *ε̄*_d-un_ − oxygen *ε*_p_

6 *γ* = CTE + CTE × coverage × (1/*N*_oxygen+coordinated metals_)

Here, *ε̄*_d-un_ represents the average unoccupied d-band center of metals coordinated with lattice oxygen, and *ε*_p_ denotes the 2p-band center of lattice oxygen. *N*_oxygen+coordinated metals_ corresponds to the total number of lattice oxygen and coordinated metal atoms considered in the CTE calculation.

Previous studies have demonstrated that atomic-scale doping significantly improves propylene selectivity.^24^ Given the high reactivity of VO_*x*_, we evaluated the catalytic performance of doped-VO_*x*_ systems. As shown in Fig. 5C, a linear relationship between *γ* and the first C–H bond activation energy is observed for doped-VO_*x*_ systems containing both dopants and Ov. Our work focused on the most active sites on different VO_*x*_ surfaces. Given our interest in the electronic effects induced by dopant metals, we developed model catalysts for the doped VO_*x*_ systems without accounting for the stepwise oxygen reduction process within the doped structures. Notably, *γ* showed moderate predictive capability for assessing C–H bond activation energies across various sites on the same given surface (Fig. S11†). Four metal dopants (W, Mo, Os, and Nb) were identified as effective in significantly reducing the reaction activity of lattice oxygen, with surface-doped sites exhibiting greater stability than subsurface and bulk-doped sites (Fig. S12†). Additionally, the Ov formation energy effectively predicts the reactivity of oxygen species,^57^ with high-valent doped VO_*x*_ exhibiting higher Ov formation energy (Fig. 5B).

While oxygen vacancies can modulate the catalytic properties of metal oxides, they also disrupt the scaling relationships seen in pristine metal oxides. A deviation in the scaling relationship between vacancy formation energy and hydrogen adsorption energy was observed (Fig. S14†), and the strong O 2p and V 3d orbital interactions near the Fermi level limit the predictive accuracy of the single O 2p-band center for C–H bond activation energy (Fig. S15†).

As depicted in Fig. 5D and S10D,† the adsorption energy of propylene associated with these dopants underwent a significant reduction, indicating that doping with high-valence metals can enhance selectivity. This change can be attributed to electronic effects, as evidenced by the observed downward shift in the O 2p-band center (Fig. S13†), while the adsorption configuration of propylene remained unchanged (Fig. S10D†). To thoroughly examine the role of dopants in regulating surface Ovc, we selected W-doped VO_*x*_ and utilized the approach outlined in the previous section to assess the oxygen vacancy distribution patterns. Fig. 5E and S16† demonstrate that, at high Ovc (above 75% ratios of oxygen vacancy) to further reduction, bulk lattice oxygen vacancies are energetically favored over the surface vacancies, forming patterns with both bulk and surface vacancies instead of further consuming surface oxygen. Notably, high-valent metal doping does not alter the oxygen carrying capacity or oxygen release (∼4.7 wt%) of vanadium-based oxygen carriers.^24^ This thermodynamically stable vacancy distribution pattern indicates an increase in lattice oxygen utilization, with more lattice oxygen being directed toward ODH rather than over-oxidation. We did not investigate kinetic differences between surface oxygen consumption and bulk oxygen diffusion rates, as our research focused on the effect of Ov on the surface reaction mechanisms. Overall, atomic-scale surface doping with high-valent metals, guided by the CTE-based descriptor *γ*, effectively regulates vacancy distribution and reduces the excessive reactivity of lattice oxygen.

## Conclusions

In summary, our work demonstrates that high-valent metal doping effectively prolongs the ODH stage of vanadium-based oxides during the CL-ODH process while improving the utilization of lattice oxygen. By systematically investigating the reaction network on surfaces with varying degrees of reduction, we revealed that based on surface oxygen vacancy coverage, the reaction process can be classified into three stages (over-oxidation, ODH and PDH). The high reactivity of lattice oxygen and the delocalized nature of vacancy effects introduce a trade-off between reaction activity and selectivity, both governed by oxygen vacancies. Using an improved charge transfer energy descriptor, we successfully screened four high-valent metal dopants (W, Mo, Nb, and Os) that significantly reduce the C–H activation capacity of lattice oxygen. These dopants also regulate oxygen vacancy distributions and stabilize surface coverage within an optimal range to extend the duration of the ODH reaction stage. In particular, defect and doping engineering can be employed to optimize vanadium-based oxygen carriers by adjusting defect concentrations through controlled oxidation time and reducing lattice oxygen reactivity to suppress over-oxidation. This work provides a strategic framework for optimizing the design of redox catalysts for CL-ODH and for predicting C–H bond activation energies across various lattice oxygen sites in complex oxide systems.

## Computational methods

We performed periodic spin polarized DFT calculations using the Vienna *ab initio* simulation package (VASP). The Perdew–Burke–Ernzerhof (PBE)^58^ exchange–correlation functional was employed and an additional coulomb repulsion parameter of *U*_eff_ = 3.2 eV (ref. 27 and 59) was applied to the d states of vanadium. Projector augmented wave (PAW)^60^ pseudopotentials were employed to describe core electrons and the valence electronic wave functions were expanded in plane waves with a cutoff energy of 400 eV. A 4 × 4 expansion of the VO_2_ (110)^61,62^ slab surface unit cell is used. The first three layers of atoms are relaxed, while the remaining two layers are fixed during the structure optimization. More details of the calculations are provided in the ESI.†

## Data availability

The data that support the findings of this study are available from the corresponding author upon reasonable request.

## Author contributions

J. L. G. and Z.-J. Z. coordinated and supervised the project. J. L. G., Z.-J. Z. and D. H. L. conceived the project idea. D. H. L. performed the DFT calculations, data processing and analysis. C. S. and X. H. W. provided suggestions from an experimental perspective. R. L., X. C. and T. Y. assisted in DFT calculations. All authors wrote and revised this manuscript.

## Conflicts of interest

There are no conflicts to declare.

## Supplementary Material

SC-016-D4SC07811J-s001
