# Supplementary material for: Role and regulation of surface oxygen vacancies in vanadium-based oxides for chemical looping oxidative dehydrogenation of propane
Source: Chem Sci. 2025 Jan 22;16(11):4710–7. doi: 10.1039/d4sc07811j (PMC11809659; doi:10.1039/d4sc07811j)
Supplement: SC-016-D4SC07811J-s001 [file SC-016-D4SC07811J-s001.pdf]

Supplementary Information (SI) for Chemical Science.
This journal is © The Royal Society of Chemistry 2025

Supporting Information

Role and Regulation of Surface Oxygen Vacancy in Vanadium-Based Oxides for Chemical Looping Oxidative Dehydrogenation of Propane

Dehui Luo[a], Ran Luo[b], Xianhui Wang[a], Xin Chang[b], Tingting Yang[b], Sai Chen[a], Zhi-Jian Zhao*[a,c], and Jinlong Gong*[a,b,c,d,e,f]

[a]*School of Chemical Engineering & Technology, Key Laboratory for Green Chemical Technology of Ministry of Education, Tianjin University; Collaborative Innovation Center for Chemical Science & Engineering (Tianjin); Tianjin, 300072, China*

[b]*Joint School of National University of Singapore and Tianjin University, International Campus of Tianjin University, Binhai New City, Fuzhou 350207, Fujian, China*

[c]*International Joint Laboratory of Low-carbon Chemical Engineering, Tianjin 300350, China*

[d]*Haihe Laboratory of Sustainable Chemical Transformations, Tianjin 300192, China*

[e]*National Industry-Education Platform of Energy Storage, Tianjin University, 135 Yaguan Road, Tianjin 300350, China*

[f]*Tianjin Normal University, Tianjin 300387, China*

*Correspondence: zjzhao@tju.edu.cn; jlgong@tju.edu.cn

Table of Contents

Computational details S3

Supplementary Figures S5

References..S21

Computational details:

Spin-polarized DFT calculations were performed using Vienna *ab initio* simulation package (VASP).[1] The Projector augmented wave (PAW) method[2] was employed to describe core electrons. The valence electronic wave functions were expanded in plane waves with a cutoff energy of 400 eV, and the convergence criteria of force and energy were set to 0.02 eV/Å and 10^{-4} eV respectively. The Perdew-Burke-Ernzerhof (PBE)[3] exchange-correlation method from generalized gradient approximation (GGA) was employed and the DFT + U method[4] was applied to the *d* states of vanadium. In this work, the recommended Ueff = 3.2 eV[5] (Ueff = U-J) was utilized. Additionally, we did not include a U correction term for the *d* states of the metal dopants following the case of the literature.[6] Dipole corrections were considered where necessary. The vacuum layer for all slab models is set to 15 Å.

The visualization of the model and electronic structure is achieved by MS and VESTA software.[7] Transition states (TS) were determined with the climbing-image nudged elastic band (CI-NEB) method and the dimer method.[8,9] Only one imaginary frequency is recognized as transition state. Atomic charges were computed using Bader charge analysis.[10] Crystal orbital Hamilton population (COHP) analysis was performed with the LOBSTER 3.2.0 package.[11,12]

A c(4x4) expansion of the VO_2 (110) slab model was chose to simulated the most stable and dominant surface.[13] The first three layers of atoms are relaxed, while the remaining two layers are fixed during the structure optimization. The k points sampling of the Brillouin zone was conducted using a Monkhorst-Pack scheme[14] of (2 × 2 × 1) for all surface optimization. For bulk optimization, k points sampling of the Brillouin zone was conducted using a Monkhorst−Pack scheme of (9 × 9 × 13). For DOS calculation, the sampling of the Brillouin zone was employed using a Monkhorst−Pack scheme of (5 × 5 × 1).

Oxygen vacancy formation energy (E_{Ov}) was calculated using the following equation:

$$E_{Ov} = E_{(1+x)\,Ov} + C_3H_6\ (g) + H_2O\ (g) - E_{xOv} - C_3H_8\ (g) \quad (1)$$

H-atom adsorption energy (E_H) was calculated using the following equation:

$$E_{H\text{-}atom} = E_{slab + H\text{-}atom} - E_{slab} - 0.5H_2\ (g) \quad (2)$$

Propylene adsorption energy was calculated using the following equation:

$$E_{C_3H_6} = E_{slab+C_3H_6} - E_{slab} - C_3H_6\ (g) \quad (3)$$

First C-H bond activation energy was calculated using the following equation:

$$E_{TS} = E_{(slab+C_3H_8)_{TS}} - E_{slab} - C_3H_8\ (g) \quad (4)$$

Oxygen 2p band center was calculated using the following equation:

$$\varepsilon_p = \frac{\int_{-\infty}^{\infty} \varepsilon p(\varepsilon) d\varepsilon}{\int_{-\infty}^{\infty} p(\varepsilon) d\varepsilon} \quad (5)$$

Unoccupied d-band center was calculated using the following equation:

$$\varepsilon_{\text{d-un}} = \frac{\int_{\text{fermi}}^{\infty} \varepsilon p(\varepsilon) d\varepsilon}{\int_{-\infty}^{\infty} p(\varepsilon) d\varepsilon} \quad (6)$$

The Gibbs free energy (G) was calculated using the following equation：

$$G = E_{DFT} + ZPE + \int_0^T C_V dT - TS \quad (7)$$

The zero-point energy (ZPE) was calculated using the following equation by VASPKIT [15]:

$$ZPE = \sum_{i=0}^{3N} \frac{h\omega_i}{2} \quad (8)$$

According high operating temperature and weak physical adsorption [T = 525 °C, $P(C_3H_8)$ = 0.23 atm], the entropies of all adsorbed state species were calculated using the following equation based on Campell-Seller's equation:

$$S_0^{ad}(T) = 0.70 S_0^{gas}(T) - 3.3R \quad (9)$$

Charge density difference of transition state for first C-H bond activation:

$$\Delta\rho = \rho(TS) - \rho(slab) - \rho(ads) \quad (10)$$

Charge density difference of defective slab:

$$\Delta\rho = \rho(defective) - [\rho(perfect) - \rho(single\ oxygen\ atom)] \quad (11)$$

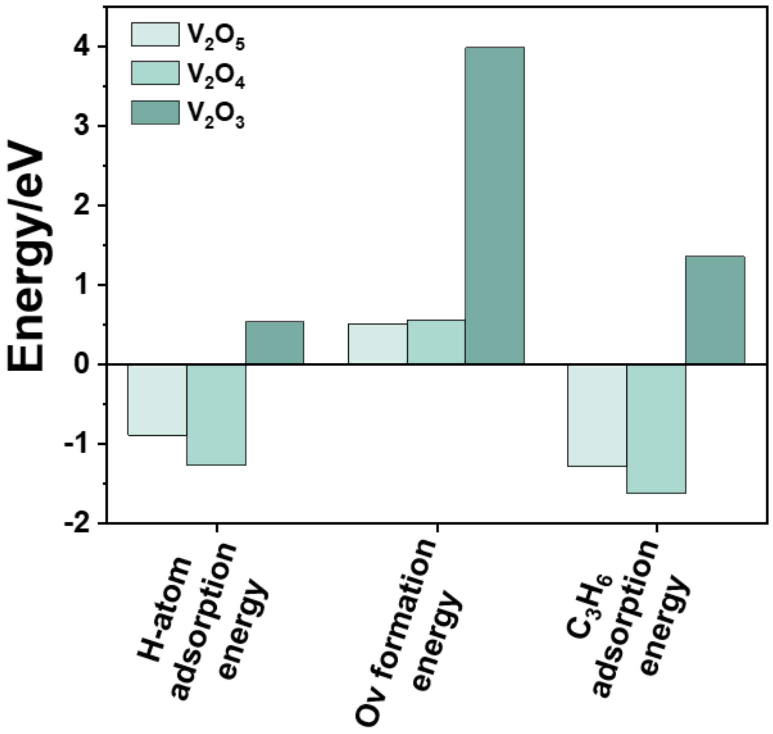

Figure S1. Comparison between different phase VO_x (V_2O_5-001, V_2O_4-110, V_2O_3-0001), H-atom adsorption energy, Ov formation energy and C_3H_6 adsorption energy.

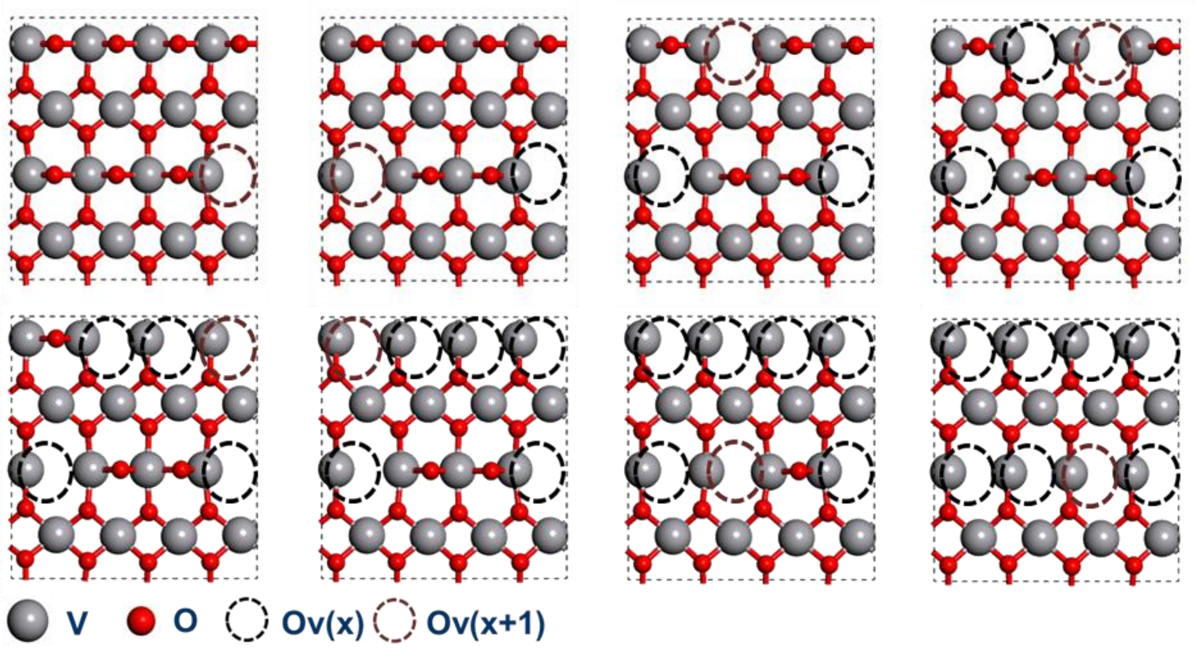

Figure S2. The locations of the surface-reducing optimum oxygen species with lowest E_{Ov} at different Ovc, from left to right, range from 0.0 Ovc to 1.0 Ovc.

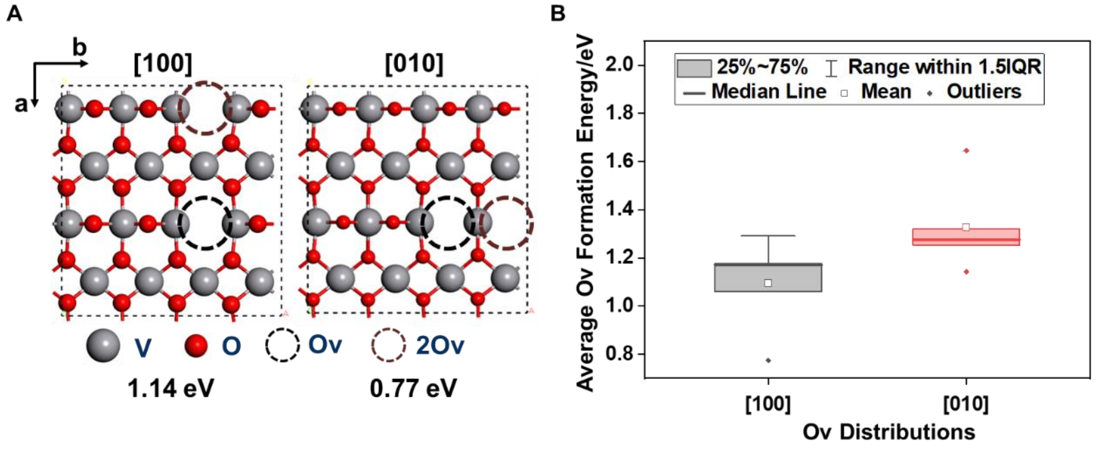

Figure S3. Comparison of Ov formation energy for different Ov distribution models at 0.25 Ovc (A). Average Ov formation energy of different Ov distribution patterns over the range of 0.00-0.75 Ovc (B).

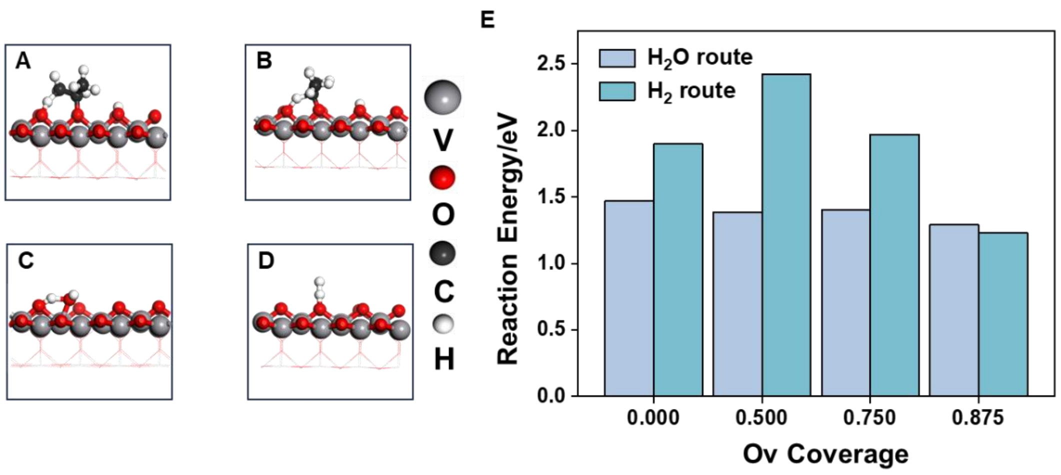

Figure S4. Transition state of C_3H_6* route (A), CH_3O*CH_3 route (B), H_2O* route(C), H_2 route (D), Plot of different Ovc surfaces against reaction energy of H_2 and H_2O* routes (E).

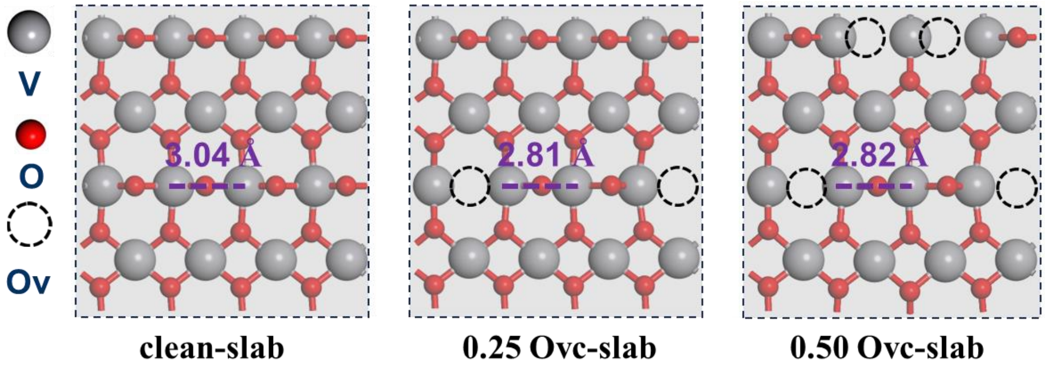

Figure S5. V-V distance between different Ovc surfaces.

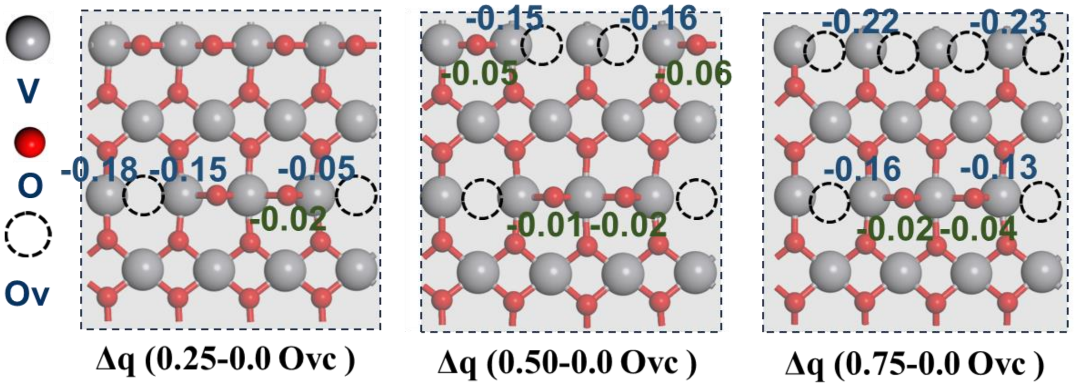

Figure S6. Bader charge difference between the 0.25, 0.50, 0.75 Ovc surfaces and the pristine surface. Green for lattice oxygen atoms, Blue for metal vanadium atoms.

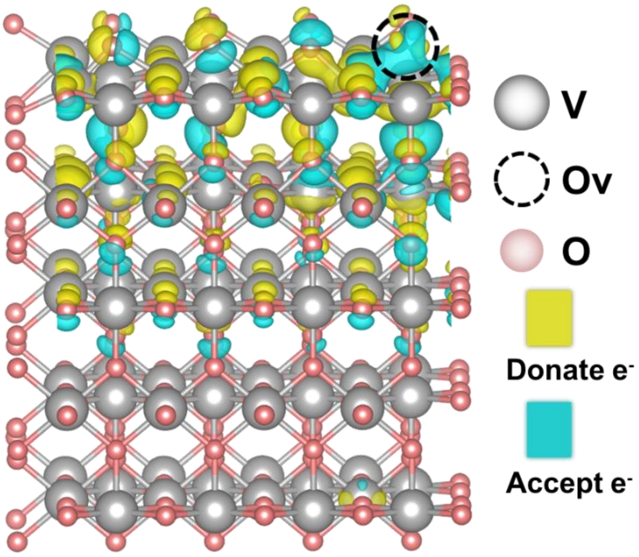

Figure S7. Charge density difference of defective surface.

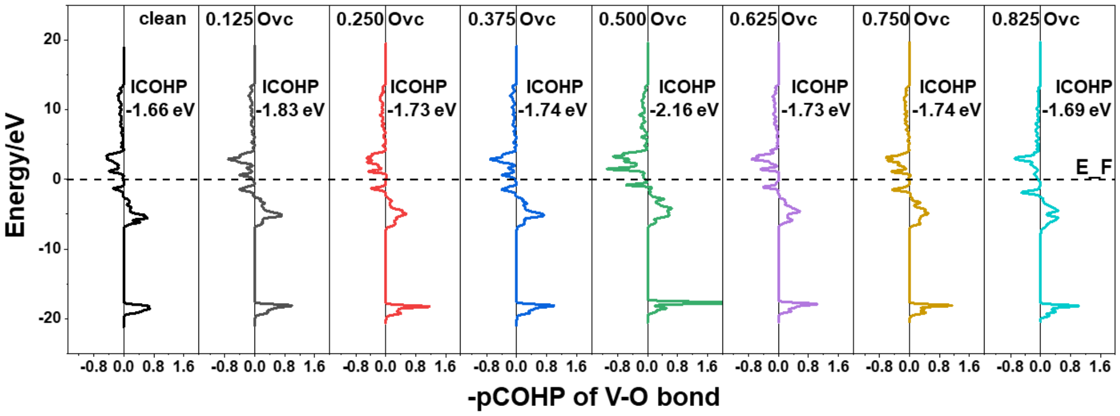

Figure S8. -pCOHP curves for V-O bonds within V-O-V species (focusing on V-O bond with higher ICOHP value) across the calculated range of Ovc surfaces.

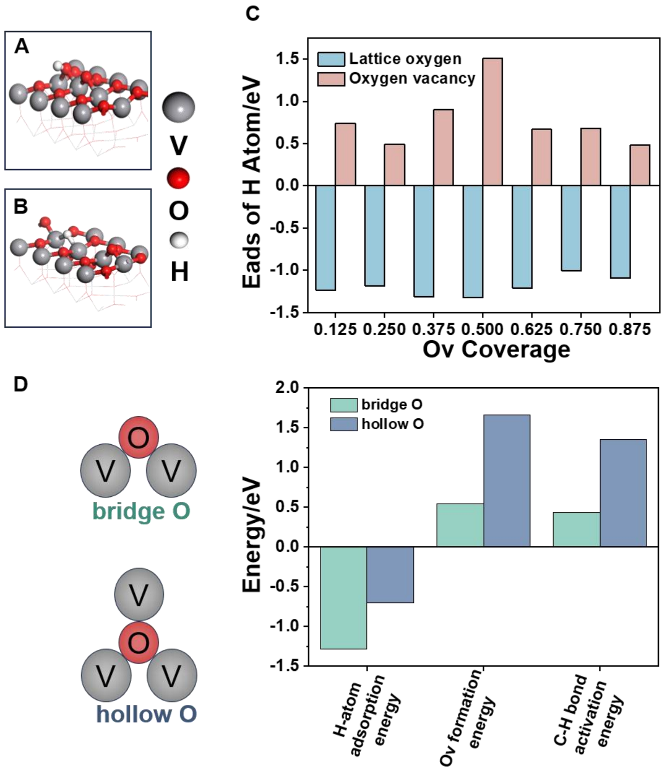

Figure S9. Hydrogen atom adsorption configuration on bridge oxygen (A), and oxygen vacancy (B). Adsorption energy of Hydrogen atom on bridge oxygen and vacancy site across VO_x surfaces with different Ovc (C). Comparison between bridge O site and hollow O site on clean VO_2 surface, H-atom adsorption energy, Ov formation energy and C-H bond activation energy (D).

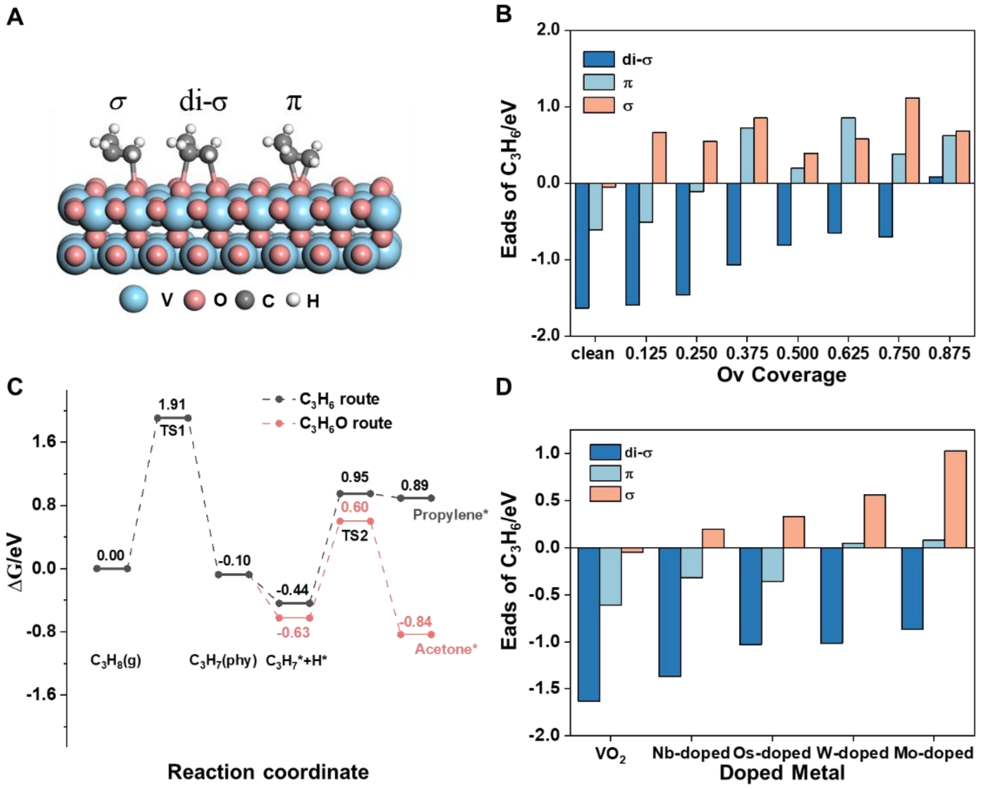

Figure S10. Adsorption modes of propylene on VO_x surfaces (A). Calculated adsorption energy (Eads) of propylene on different Ovc surfaces (B). Reaction pathway and calculated Gibbs's free energy [T = 525 °C, P (C_3H_8) = 0.23 atm] for propylene and acetone route (C). Calculated adsorption energy (Eads) of propylene on different metal-doped VO_x surfaces (D).

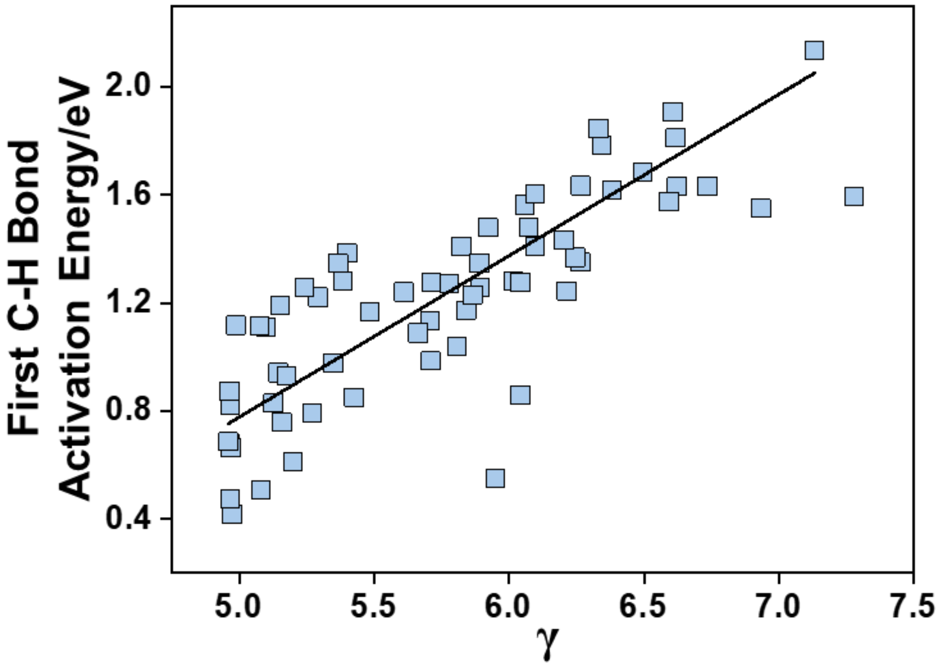

Figure S11. Plot of First C-H bond activation energy against improved parameter γ on VO_x surfaces with different Ovc and metal-dopants.

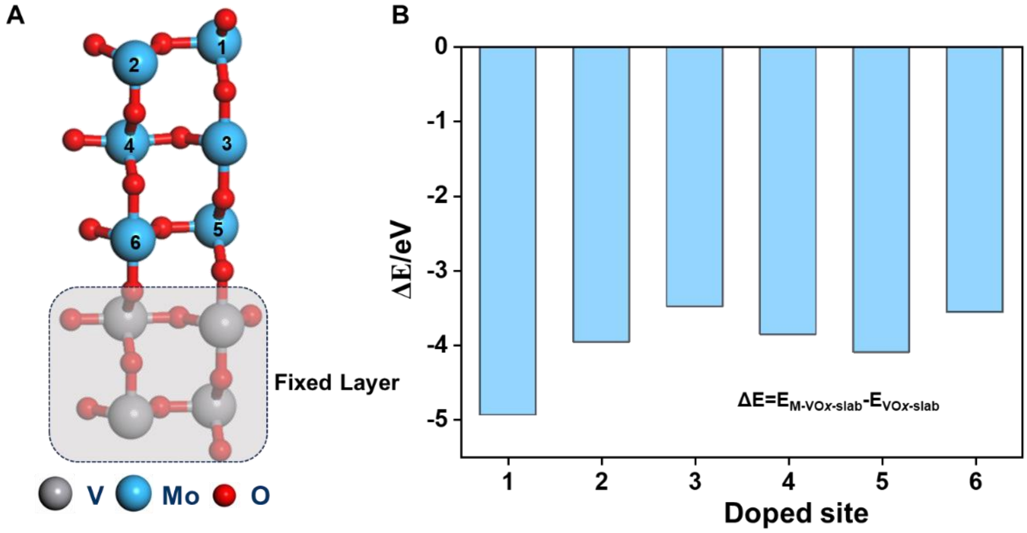

Figure S12. Mo doped VO_x models on different sites (A). Difference energy of different Mo doped sites refer to pristine surface (B).

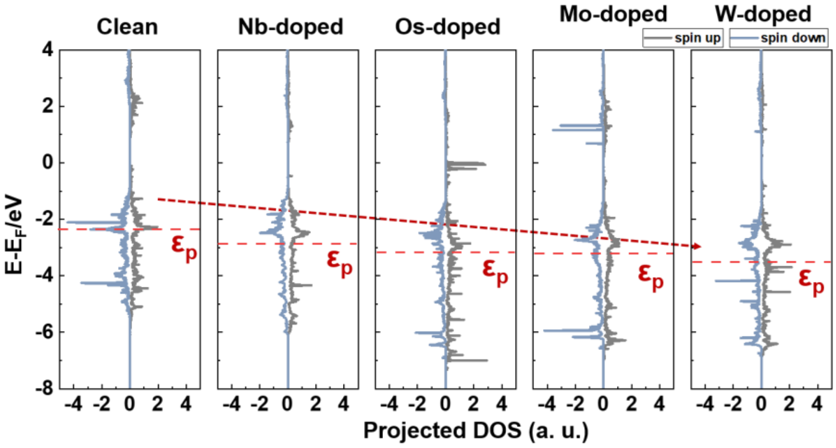

Figure S13. DOS (Density of state) projected onto the p-bands of surface oxygen atoms on the clean, Nb-doped, Os-doped, Mo-doped, W-doped surfaces (spin up and down). E_F is the fermi level and ε_p is the average O-2p band center of spin up and down.

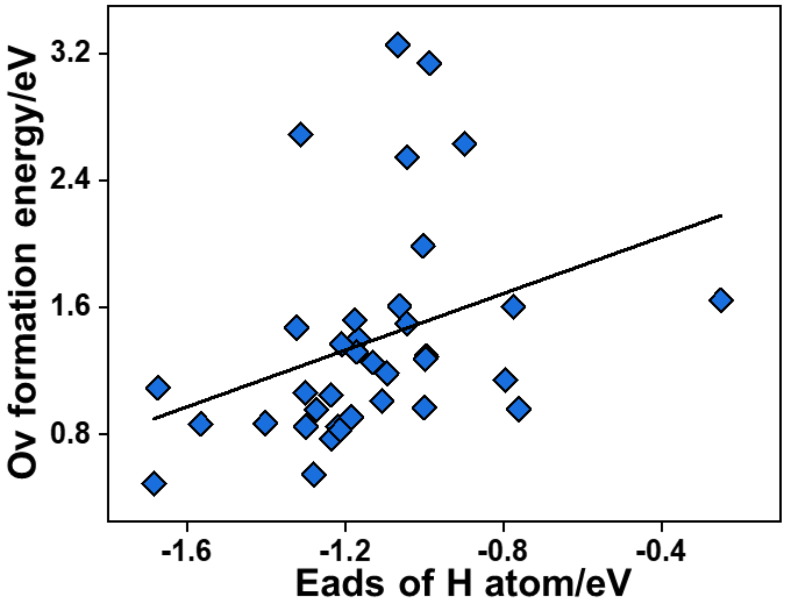

Figure S14. Plot of Hydrogen atom adsorption energy on bridge oxygen against Ov formation energy with different Ovc on VO_x surfaces.

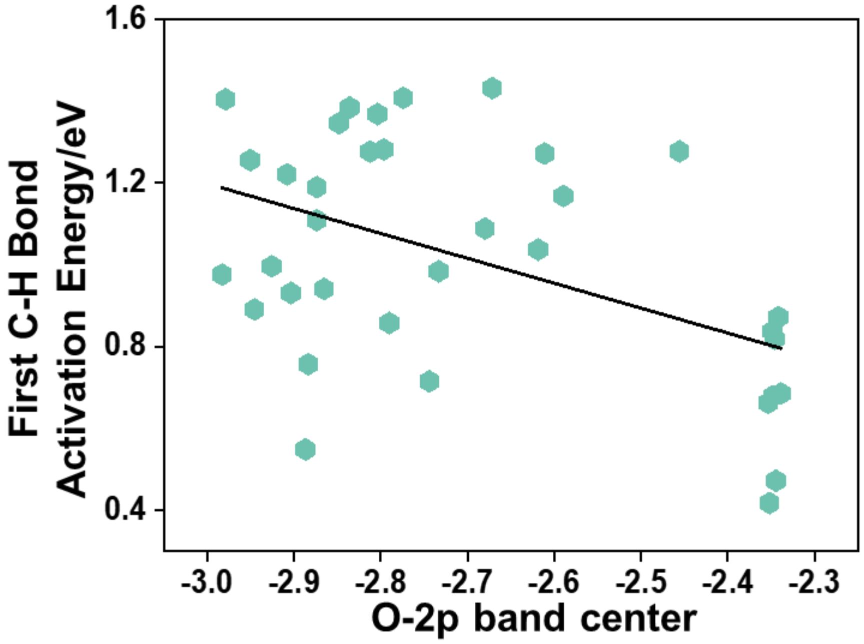

Figure S15. Plot of First C-H bond activation energy against O-2p band center on VO_x surfaces with different Ovc.

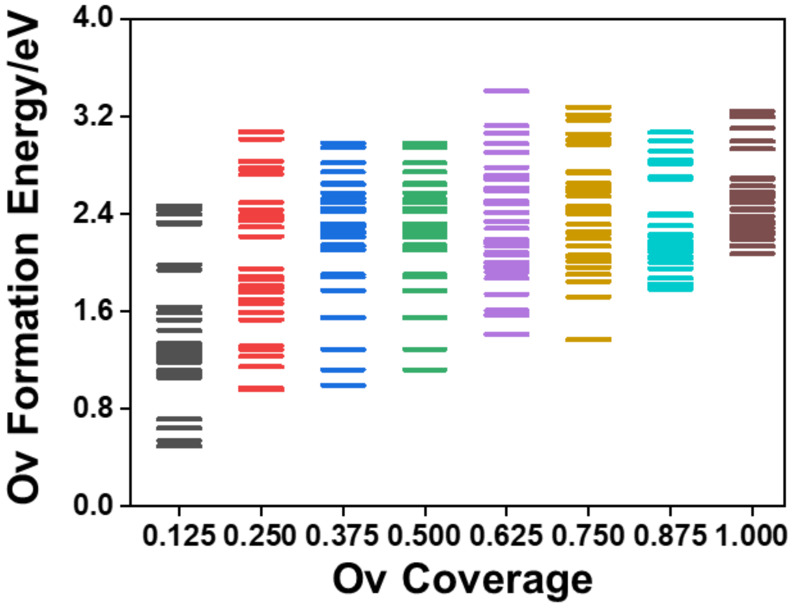

Figure S16. Ov formation energy for various Ov distribution patterns with different Ovc in W-doped VO_x surface.

References:

1 G. Kresse and J. Furthmüller, *Phys. Rev. B*, 1996, **54**, 11169–11186.

2 G. Kresse and D. Joubert, *Phys. Rev. B*, 1999, **59**, 1758–1775.

3 J. P. Perdew, K. Burke and M. Ernzerhof, *Phys. Rev. Lett.*, 1996, **77**, 3865–3868.

4 V. I. Anisimov, J. Zaanen and O. K. Andersen, *Phys. Rev. B*, 1991, **44**, 943–954.

5 L. Wang, T. Maxisch and G. Ceder, *Phys. Rev. B*, 2006, **73**, 195107.

6 M. D. Krcha, A. D. Mayernick and M. J. Janik, *J. Catal.*, 2012, **293**, 103–115.

7 K. Momma and F. Izumi, *J. Appl. Crystallogr.*, 2011, **44**, 1272–1276.

8 G. Henkelman, B. P. Uberuaga and H. Jónsson, *J. Chem. Phys.*, 2000, **113**, 9901–9904.

9 G. Henkelman and H. Jónsson, *J. Chem. Phys.*, 1999, **111**, 7010–7022.

10 R. F. W. Bader, *Chem. Rev.*, 1991, **91**, 893–928.

11 R. Dronskowski and P. E. Bloechl, *J. Phys. Chem.*, 1993, **97**, 8617–8624.

12 S. Maintz, V. L. Deringer, A. L. Tchougréeff and R. Dronskowski, *J. Comput. Chem.*, 2016, **37**, 1030–1035.

13 H. Yuan, J. Chen, H. Wang and P. Hu, *ACS Catal.*, 2018, **8**, 10864–10870.

14 H. J. Monkhorst and J. D. Pack, *Phys. Rev. B*, 1976, **13**, 5188–5192.

15 V. Wang, N. Xu, J.-C. Liu, G. Tang and W.-T. Geng, *Comput. Phys. Commun.*, 2021, **267**, 108033.
